# Contact Effect Contribution to the High Efficiency of Lithium Chloride Against the Mite Parasite of the Honey Bee

**DOI:** 10.3390/insects11060333

**Published:** 2020-05-28

**Authors:** Éva Kolics, Kinga Mátyás, János Taller, András Specziár, Balázs Kolics

**Affiliations:** 1 Department of Plant Sciences and Biotechnology, Georgikon Faculty, University of Pannonia, H-8360 Keszthely, Hungary; kolicseva@gmail.com (É.K.); petrovicsnemkk@gmail.com (K.M.); taller@georgikon.hu (J.T.); 2 Balaton Limnological Institute, Centre for Ecological Research, H-8237 Tihany, Hungary; specziar.andras@okologia.mta.hu

**Keywords:** lithium chloride, contact mode of action, *Apis mellifera*, *Varroa*

## Abstract

Lithium chemicals have been proven to be very effective in eradicating *Varroa destructor*, the detrimental parasite of the honey bee; however, little is known about the side effects on brood and long term consequences on the colony. Earlier, it was proposed that the action mechanisms of lithium chloride do not include the contact mode. Here, we investigate this question using a paper strip test to demonstrate the concentration-dependent effectiveness of lithium in the contact mode of action, confirming that it is also a contact agent against the *Varroa* mite. According to our knowledge, this is the first report on the high varroicidal effect of lithium in the contact mode of action. Our findings may open up possibilities for novel ways of treatment (e.g., the use of lithiated strips) in the event that lithium salts become legal for use in apiculture.

## 1. Introduction

Maintenance of commercial honey bee (*Apis mellifera*) colonies is highly dependent upon the successful control of the parasitic mite *Varroa destructor*, recognized as the biggest threat to the western honey bee worldwide. Left untreated, mites can kill an entire colony within one or two years [1,2]; however, in areas of high bee density, it may occur within an apicultural season. Controls can be effective and of low risk of building resistance [3,4,5,6], but in some countries, they are restricted mainly to only a few chemicals, implying the potential development of acaricide resistance [7,8] and reducing the possibility of mite eradication in the foreseeable future. Parallel to this, there is an increasing demand to avoid the build-up of miticide residues or their metabolites in honey and wax. Alongside novel RNAi-based approaches [9], it was observed that lithium salts may offer promising and easy-to-use chemicals for effectively treating *Varroa* infestation. Furthermore, treatments have been published where 100% mite mortality was found in the brood-free period with minor or no mortality of adult bees, with certain concentrations of lithium-containing chemicals [10,11]. Lithium chloride has been described as a varroicide that acts in a systemic mode of action in a wide range of concentrations [10]. High miticidal activity was exerted in artificial swarms applying 25 and 50 mM lithium chloride in sugar syrup and patties, respectively [12,13]. 

Based on earlier unpublished attempts where lithium chloride showed high effectiveness at very low concentrations, we supposed that it might have an additional effect in a contact mode of action. The aim of this study was to test this hypothesis with insights from in situ application to enable demonstration in commercial bee colonies.

## 2. Materials and Methods

**Figure 1:**
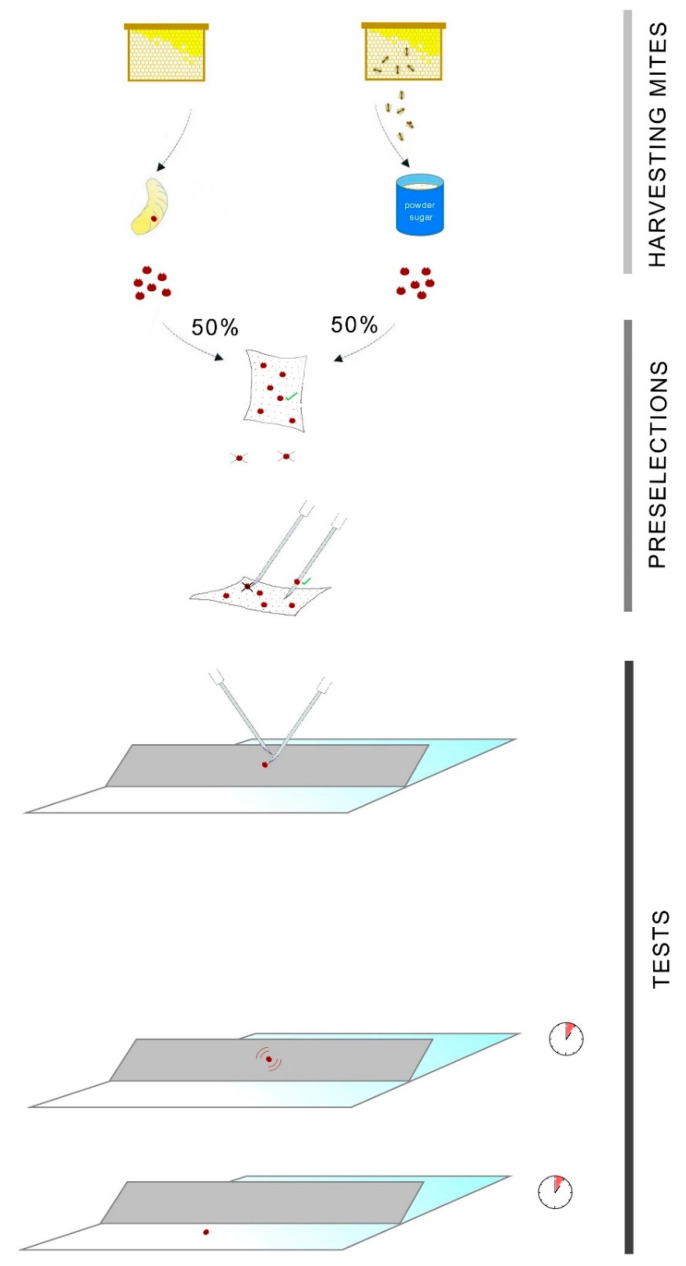
Overview of the experimental design. Mites collected from sealed brood cells using powder sugar were preselected to keep only vital individuals, discarding mites unable to grasp a paper towel. Subsequently, mites were placed on the experimental paper strips one at a time with the help of sterile syringe needles, using only mites willing to climb onto them. Paperboard impregnated with lithium chloride solution was fixed on a flat glass surface at a 45° angle, while control strips were impregnated with deionized water. One mite was placed onto each lithiated strip and another mite onto the control strip at the same time. After initiation, the first event recorded was the onset of tremorous, uncontrolled movements. The second recorded event was when the mite fell off the strip, considered to be the miticidal threshold.

Adult mites were freshly obtained from sealed brood cells and collected using a powder sugar test of heavily infested *Apis mellifera carnica* colonies. Mites were placed onto a vertical paper towel using a fine brush. To preselect vital individuals, mites that were unable to grasp strongly for about 30 min were discarded (6%). The remaining mites were kept at 25 °C for a maximum of 120 min in order to prevent a decrease in vitality and mobility. Subsequently, mites were placed on experimental paper strips one at a time with the help of sterile syringe needles. While transferring specimens from the paper towel, an additional pre-selection was implemented, as only mites willing to climb onto the needle by themselves were used (96%). Experimental paperboard strips (1.5 mm in thickness, 3 cm × 20 cm in area) were evenly impregnated with 2 mL lithium chloride solution (LiCl 1H_2_O) of one of the 11 tested concentrations ranging from 10.78 mM to 11.04 M (10.78, 21.55, 43.11, and 86.22 mM, and 0.17, 0.34, 0.69, 1.38, 2.76, 5.52, and 11.04 M). The strips were fixed on a flat glass surface at a 45° angle. Control strips were impregnated with deionized water from the same stock that we used for preparing the lithiated solutions. One mite was placed onto each lithiated strip and another mite onto the control strip at the same time (the number of individuals used for each exposure varied between three and 11; the total number of mites used was 71 each for the treated and control groups). After initiation, the first event recorded was the onset of tremorous movements accompanied by uncontrollable movements. The second recorded event was when the mite fell off the strip, which we considered to be the miticidal threshold. For overview of the in vitro experimental design, see Figure 1.

The concentration-dependent contact effect of LiCl on the log10 transformed time of the first tremorous movement and of the drop of the mites was evaluated by analysis of variance (ANOVA) followed by Tukey HSD post hoc tests. Levene’s test for the homogeneity of variance of data (F_10;60_ = 1.45, *p* = 0.180 for time to first tremorous movement and F_10;60_ = 1.65, *p* = 0.114 for time to drop) and the Kolmogorov–Smirnov test of normality on residuals (N = 71, D = 0.103, *p* = 0.405 for time to first tremorous movement and N = 71, D = 0.109, *p* = 0.345 for time to drop) proved that the assumptions of the ANOVA were met. Since none of the control mites showed any tremorous movements or dropped down from the experimental paperboard during the 120 min of the observation period, the control groups could not be included in the ANOVA test. Therefore, the Z-test was used to compare the proportion of mites that responded (i.e., showed tremorous movement and dropped) between the control and LiCl treatments.

In order to demonstrate that lithiated strips show in situ effectiveness, strips were prepared by impregnating each strip with the amount used for a trickling dose for one hive (2.28 mL 5.52 M LiCl 1 H_2_O = 0.76 g LiCl 1 H_2_O). This demonstration was carried out in three broodless commercial bee colonies, in the pre-wintering period in November 2019, at Keszthely, Hungary (GPS: 46°45′55.6″ N, 17°14′52.6″ E), registering the number of mites counted on the sticky board. Commercial colonies were selected according to their previous mite fall; we picked these colonies from the most infected ones of an apiary of 120 colonies, based on the mite fall carried out using Apivar strips at the end of September in the broodright stage. At that time, mite fall was 116, 102 and 67 for hives No. 1, 2 and 3, respectively. Additionally, in all of these colonies, bees with mites on the thorax could be observed in October, suggesting an elevated level of infestation. The colonies had not been treated from September until the start of the experiment in order to preserve the mites. Experimental hive No. 1, used as control, was left untreated until the end of the trial. In hives No. 2 and 3, treatment was started with one lithiated strip placed in the middle of the nest. Colonies were broodless in order to assure that the varroicide effect was not influenced by the addition of hive-born mites. After tracking the effect for five days by counting mites, five additional strips were inserted and the recording of mite fall was continued. Finally, all three hives were controlled by trickling lithiated syrup (40 mL, 250 mM) at the same time.

## 3. Results

**Figure 2:**
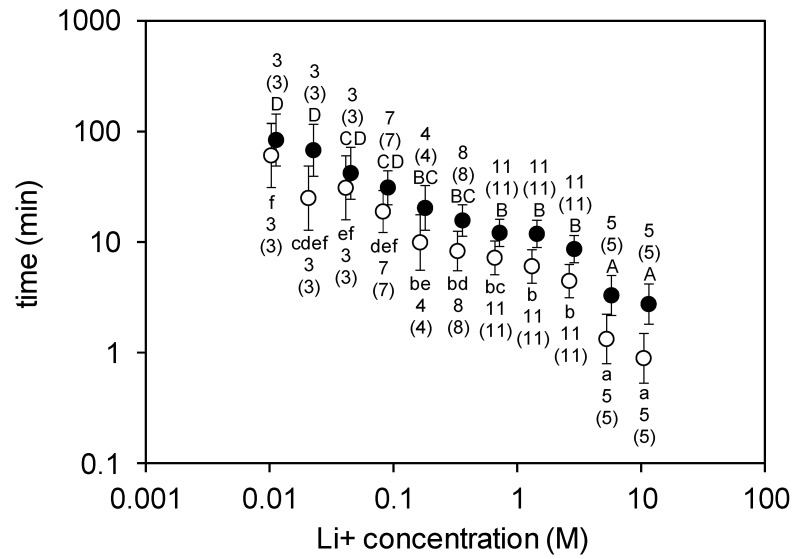
Analysis of variance (ANOVA) showed a concentration-dependent response of mites to lithium chloride (Li+) contact treatment, as monitored by time (mean ± 95% CI) to first tremorous movement (○; F_10;60_ = 21.2, *p* < 0.001, log10 transformed data) and time to drop (●; F_10;60_ = 21.2, *p* < 0.001, log10 transformed data) from the treated paperboard strip. Plotted values not sharing any letter (lowercase for time to first tremorous movement and uppercase for time to drop) are statistically different at *p* < 0.05 (Tukey HSD post hoc test). The number of mites tested in the treated and control groups and the number of mites that responded (in brackets) to the treatment are indicated. Since none of the control mites responded (neither showed tremorous movements nor dropped) during the 120 min of the observation period (time to first tremorous movement > 120 and time to drop > 120), the values for the control mites could not be plotted. Note that in order to improve the readability of the graph, the data on time to first tremorous movement and time to drop are shown slightly offset to the side of the actual Li+ concentration.

In the paper strip-based contact tests, mites on control strips either remained immobile (*n* = 58) or moved away by a maximum of 2 cm on the surface (*n* = 12); none of them fell down or showed similar signs to those on treated strips (Z = 11.9, *p* < 0.001 for both tremorous movement and drop between control and LiCl-treated groups). On the paper strips impregnated with increasing concentrations of lithium chloride, the duration of time until the first appearance of tremorous movements in the mites became progressively shorter. At the lowest concentration (10.78 mM), symptoms appeared about after 66 min in average and mites fell down after 84 min. At the highest concentration (11.04 M), mites started to show tremorous symptoms within about a minute and fell down in the third minute of the trial on average (Figure 2). It is worth noting that none of the treated mites that fell off the strip recovered from the lithium-caused symptoms at any of the lithium concentrations tested. Their tremor tended to fade gradually into uncontrollable movement and they finally ended up immobile. Every mite exposed to lithium was first affected by tremor and subsequently dropped. Only the time that passed until the onset of tremor and mite fall showed differences according to concentration.

In the two commercial colonies (No. 2 and 3), which were used to test whether a contact effect can be achieved in situ, 24 and 6 mite falls were detected during the first five days of the treatment carried out by placing a single lithiated strip into each hive. At the same time, in the control hive (No. 1), only three mite falls were registered. The number of mite falls significantly increased in the second phase, when we inserted an additional five strips into each of the two treated hives and left them for 10 days (without replacement or refreshment of the previous strip). This treatment resulted in a fall of 198 and 41 mites for hives No. 2 and 3 respectively, whereas only four mites fell in the control hive during the same period.

Finally, the application of trickling lithiated syrup at the end of the 15th day yielded additional falls of 18, 5 and 120 individuals in hives No. 2, 3, and 1 (control) after 10 days respectively. A subsequent powder sugar test carried out on 100 g of bees yielded no mites in any of the hives (a summary of the results can be found in Table 1). The symptoms of the freshly fallen mites under the treatment carried out with both lithiated strips and trickling were identical to those observed in the contact test. Applying a strip test to the commercial colonies confirmed that the contact mode of action may be exerted in situ at certain concentrations.

## 4. Discussion

The first publication on the varroicidal effect of lithium salts revealed that they have high effectiveness with a systemic mode of action, exerting 100% mite mortality and low toxicity to adult bees, in artificial swarms already at a concentration of 25 mM [10,11].

Here we present novel results proving that LiCl also has a strong contact effect on mites. This finding widens the range of the potential applicability of this compound as a miticide in honey bee colonies. Moreover, contact tests with impregnated paper strips revealed the irreversible effect of LiCl on mites even at very low concentration. It should be noted that the lithium concentration of the trickling solution may have implications for its two-level action (i.e., systemic and contact) as well. 

The lowest tested concentration (10.78 mM) of LiCl solution was set according to the requirement that impregnated strips should remain wet for the whole period of treatment. This criterion was set because we assume that the penetration of lithium to the parasite is effective only in solution. The lithiated strips remained wet at the highest concentration (11.04 M) for at least 15 days both in vitro and in situ, revealing that LiCl might not need any additional humectant for long-term contact treatment in cases where this concentration is used on strips.

The application of LiCl as a contact treatment may represent a beneficial alternative to feeding it to bees and can utilize its systemic mode of action in mite control. Contact treatments might imply a possible lowered risk of honey contamination [14], compared with previous approaches where lithium was administered via feeding [10,11]. Our investigations were restricted to the pre-wintering, brood-free stage of bee colonies. Although post-experiment powder sugar tests did not yield any mites, since only 100 g of bees were tested in each hive (according to the protocol of breeders of AGT, Germany [15]), colonies might have had remaining mites, but it is likely that they did not meet the harmful threshold. Furthermore, methodological restrictions inevitably accompany this pilot study, limiting our knowledge of the efficacy and eventual side effects of LiCl during the reproductive season. 

## 5. Conclusions

To conclude, as well as the previously verified systemic mode of action [10], LiCl clearly has a contact effect on mites, and this feature may significantly extend the range of its applicability. The bifold mechanisms of lithium chloride in mite control may support lithium as a promising tool for controlling mite infection if it becomes a registered varroicide. However, further research is needed to determine the concentration and the most optimal method with regard to its efficacy, which may also depend on temperature and humidity conditions. Moreover, detailed examination is required to quantify the eventual side effects on brood.

## Figures and Tables

**Table 1:** In situ testing resulted in a high number of mites on the sticky board.

Hives	Control (No. 1)	No. 2	No. 3
Mite fall after Apivar treatment 1.5 months before the trial	116	102	67
One strip for 5 days (beginning of day 1)	3	24	6
Additional 5 strips inserted (end of day 5)	4	198	41
Trickling lithiated syrup (end of day 15)	120	18	5
Powdered sugar test (day 15)	0	0	0
